# pH-Sensitive Poly(acrylic acid)-g-poly(L-lysine) Charge-Driven Self-Assembling Hydrogels with 3D-Printability and Self-Healing Properties

**DOI:** 10.3390/gels9070512

**Published:** 2023-06-25

**Authors:** Maria-Eleni Kargaki, Foteini Arfara, Hermis Iatrou, Constantinos Tsitsilianis

**Affiliations:** 1 Department of Chemical Engineering, University of Patras, 26500 Patras, Greece; kargakim@upnet.gr; 2 Department of Chemistry, University of Athens, Panepistimiopolis, Zografou, 15771 Athens, Greece; a.foteini@gmail.com (F.A.); iatrou@chem.uoa.gr (H.I.)

**Keywords:** hydrogel, poly(acrylic acid)-*g*-poly(L-lysine), self-assemble, 3D-network, shear-induced 3D printability, pH sensitivity, PLL secondary structure

## Abstract

We report the rheological behavior of aqueous solutions of a graft copolymer polyampholyte, constituted of polyacrylic acid (PAA) backbone grafted by Poly(L-lysine) (PAA-*b*-PLL). The graft copolymer self-assembles in aqueous media, forming a three-dimensional (3D) network through polyelectrolyte complexation of the oppositely charged PAA and PLL segments. Rheological investigations showed that the hydrogel exhibits interesting properties, namely, relatively low critical gel concentration, elastic response with slow dynamics, remarkable extended critical strain to flow, shear responsiveness, injectability, 3D printability and self-healing. Due to the weak nature of the involved polyelectrolyte segments, the hydrogel properties display pH-dependency, and they are affected by the presence of salt. Especially upon varying pH, the PLL secondary structure changes from random coil to *α*-helix, affecting the crosslinking structural mode and, in turn, the overall network structure as reflected in the rheological properties. Thanks to the biocompatibility of the copolymer constituents and the biodegradability of PLL, the designed gelator seems to exhibit potential for bioapplications.

## 1. Introduction

The macromolecular design has been established as a molecular tool to create three-dimensional networks formed by the self-organization of polymeric materials through various reversible interactions, forming weak non-covalent bonds. These networks swollen in aqueous media form soft materials, the so-called hydrogels, that have attracted immense attention due to a large variety of potential applications [1,2,3,4,5]. The incorporated functionalities of the polymers involved in the network determine the interactions, i.e., hydrophobic, ionic (also called electrostatic), hydrogen-bonding, host-guest and coordination complex, that develop the physical crosslinks [6]. The bond energies of the physical crosslinks affect the dynamics of the networks. Another important factor that must also be considered in the hydrogel design is the macromolecular architecture, e.g., linear, star and graft block copolymers, including a degree of polymerization of the various segments, that affect the overall structure of the network [7]. All these factors affect the hydrogel’s macroscopic properties, for instance, porosity, injectability, printability and self-healing, which are crucial for their specific applications. Thus, the rational design of the polymer gelator allows tailoring the macroscopic properties of the hydrogels for targeting applications.

The charge-driven assembly concept has been used to design networks based on interactions between oppositely charged moieties, the bond energy of which ranges from 5 to 200 KJ/mol [6]. Synthetic and natural polyelectrolytes and/or polyampholytes, bearing charged and/or pH-dependent ionogenic functionalities on their repeating monomer units are the basic constituents of such networks, which are developed by physical crosslinking through ionic interactions [8]. Several strategies have been developed depending on the crosslinking mode of the macromolecules, that is, self-assembly of polyampholytes, where the opposite charges located in the same macromolecule either in random [9] or in block monomer distribution [10,11,12,13], co-assembly of negatively and positively charged polyelectrolytes [14,15,16,17,18,19,20,21,22] and polyelectrolytes complexed with multivalent ions [23,24].

Peptide-based hybrids constituted of low molecular weight polymers and short peptides have been designed as supramolecular gelators [25,26,27]. Synthetic polypeptides, on the other hand bearing pendant weak ionic groups (e.g., COOH, NH_2_), exhibiting thus the pH-dependent degree of ionization, are very interesting candidates as charged segments for designing macromolecules capable of forming networks through ionic complexation [25,28,29]. The advantage of incorporating polypeptides into the hydrogel network is that it is endowed with biocompatibility and biodegradability, which is required for specific biomedical applications. Another interesting future of polypeptides is that they exhibit secondary structures (i.e., random coil, *α*-helix and β-sheet), which undergo transitions upon varying the pH of the medium [30]. As it is well known, the pH-dependent secondary structures affect the self-assembly behavior and the rheological properties of peptide-based hydrogels [31,32,33]. An interesting strategy to attain physically ionic crosslinked hydrophilic biodegradable 3D networks using oppositely charged polypeptide segments has been proposed recently [34]. In that work, two ABA triblock copolymers with a common poly(ethylene glycol) (PEG) middle block and different outer blocks of poly(glutamic acid) and poly(L-lysine) (PLL) of nearly similar degrees of polymerization were mixed to obtain free-standing gels in aqueous media. The formed 3D network is constituted of polypeptide ionic complexes (coacervate domains) bridged by the PEG middle blocks. The hydrogel properties could be tuned by the polymer composition and concentration and were dependent on the pH and ionic strength of the medium. Those peptide-based hydrogels exhibit good cytocompatibility and biodegradability.

In the present work, an alternative strategy is proposed by using a single graft copolymer as a gelator, constituted of a long negatively charged poly(acrylic acid) (PAA) grafted by a number of short positively charged poly(L-lysine)s. The PAA-*g*-PLL self-assembles spontaneously in aqueous media, forming a 3D network. The high stochiometric asymmetry (PAA/PLL 92/8 mol%) of the oppositely charged segments ensures the formation of a water-soluble swellable network. A free-standing hydrogel is formed above a percolation threshold determined at about 2.35 wt% polymer concentration. The mechanical features of the hydrogels were studied by steady-state and oscillatory rheological experiments. Due to the weak nature of the involved polyelectrolyte segments, the hydrogel properties display pH-dependency, and they are affected by the presence of salt. More importantly, the hydrogel exhibits shear-induced 3D printability and self-healing properties. Worthy noted that the precursor graft copolymer bearing the same number of grafted hydrophobic poly(boc-LL) (PAA-*g*-P(boc-LL)) forming hydrophobically crosslinked hydrogels were recently reported [35]. Thus, it gives us the opportunity to discuss the effect of different association modes (ionic vs. hydrophobic interactions) on the structure and mechanical response of the two hydrogel formulations.

## 2. Results and Discussion

**Scheme 1:**
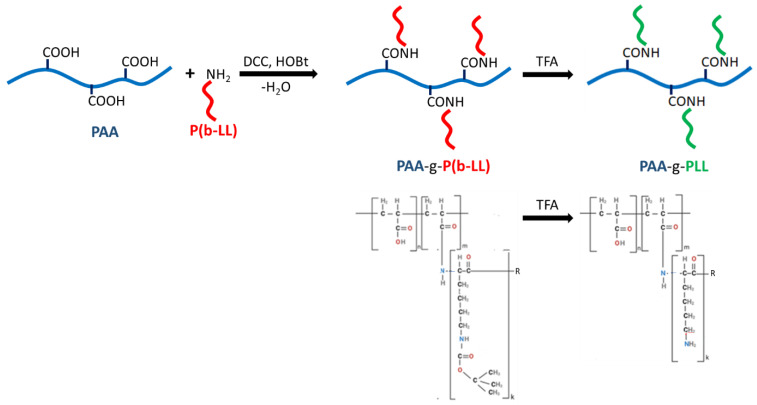
The reaction scheme of the PAA-*g*-PLL synthesis.

The graft copolymer under investigation was synthesized in a two-pot reaction. Firstly, PAA was grafted by PLL using standard carbodiimide chemistry by reacting -NH_2_ of the N-termini of the protected P(boc-LL) with the pendant -COOH of the PAA, forming amide bonds. Secondly, the side chains of PAA-*g*-P(boc-LL) were deprotected by hydrolysis using trifluoroacetic acid (TFA), yielding the PAA-*g*-PLL (Scheme 1).

### 2.1. Self-Assembling Hydrogel

The PAA-*g*-PLL graft copolymer is constituted of a long PAA backbone (mean degree of polymerization 6250) bearing about 11 PLL side chains (mean degree of polymerization 50). Indeed, it is a block-type highly asymmetric weak polyampholyte bearing oppositely charged ionogenic -COOH (*pKa*~4.5) and -NH_2_ (*pKa*~10.8) moieties, which are charged by deprotonation protonation, respectively, depending on the environmental pH. Preliminary rheological comparative investigations were conducted between the PAA precursor and the PLL-grafted one by rheology. Steady-state shear experiments of 5 wt% aqueous solutions (see Figure S1 in Supplementary Materials) showed that the low shear viscosity of the graft copolymer increased more than three orders of magnitude with respect to its PAA precursor. Moreover, strain sweep oscillatory data of the same formulations showed that the graft copolymer solution exhibits gel-like behavior since the storage modulus G′ is higher than the storage modulus G″ in contrast to the PAA formulation (Figure S1). The pH of solutions was regulated to physiological 7.4, where both the anionic and cationic blocks of the graft copolymer are nearly fully charged. Thus, it is suggested that the solution’s strong thickening phenomenon is due to the self-assembly of the copolymer chains by ionic complexation of the oppositely charged PAA/PLL blocks forming a 3D network structure.

**Figure 1:**
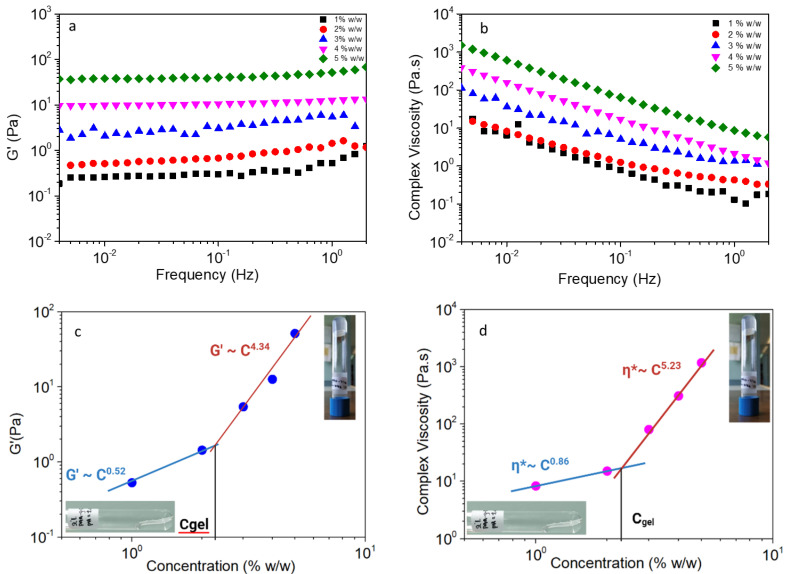
Angular frequency dependence of (**a**) storage modulus (G′) and (**b**) complex viscosity of PAA-*g*-PLL aqueous solutions at various concentrations, pH = 7.4, fixed strain amplitude = 1% (LVR) and temperature 25 °C. Storage modulus (at angular frequency 1 Hz) (**c**) and complex viscosity (at angular frequency 0.005 Hz) (**d**) as a function of concentration at a fixed strain amplitude of 1% (LVR), pH = 7.4 and temperature 25 °C. The digital photos (inset of c, d) indicate the transformation of the weak viscoelastic solution to free-standing gel by increasing the concentration of PAA-*g*-PLL.

To estimate the percolation threshold of the network structure, PAA-*g*-PLL aqueous solutions were prepared at physiological pH 7.4, with polymer concentrations (C_P_) of 1–5 wt% and explored by oscillatory shear rheology at room temperature. Figure 1a,b depicts the frequency dependence of storage modulus G′ and the complex viscosity η* at various polymer concentrations. G′ predominates G″ (Figure S2) at the low-frequency range in all polymer concentrations. The frequency dependence is very weak, and η* decreases with frequency without the appearance of a plateau in the low-frequency range, implying gel-like behavior. However, optical observations showed that the formulations flow at low concentrations and form free-standing gels at elevated C_P_ (inset of Figure 1d). In Figure 1c,d, G′ and η* (at a fixed frequency) have been plotted as a function of C_P_. Both factors increase with concentration, initially smoothly and above a critical concentration very abruptly. At the intersection of the two lines, a critical polymer concentration, at about 2.35 wt%, is denoted as C_gel_, provided that above it, the formulations form free-standing gels.

At C_P_ < C_gel,_ the solutions exhibit weak viscoelasticity (G′ > G″, with values below 1 Pa) even at low frequencies, which should be mainly attributed to chain entanglements. We assume that below C_gel,_ the interactions between the oppositely charged segments of the graft copolymer led mainly to intramolecular associations rather than to intermolecular ones. Indeed, viscosity data of the PAA precursor (see Supplementary Materials) revealed that polymer solutions are in the entangled regime (C_p_ > C_e_). This is also corroborated by the fact that the low shear viscosity for all solutions is higher than that typically reached at the entanglement concentration C_e_, i.e., η ≈ 30η_solvent_ ≈ 0.03 Pa·s [36]. Above C_gel_, intermolecular ionic interactions between oppositely charged PAA/PLL segments predominate, forming polyelectrolyte complexes acting as crosslinks of a percolated network. The storage modulus and the complex viscosity of the hydrogels scale with power laws exhibiting exponents of 4.34 and 5.23, respectively (Figure 1c,d). These high values occurring above the percolation threshold seem typical for associating hydrogels [37]. In the same concentration regime, the exponent of the viscosity power law for the PAA precursor in salt-free aqueous solutions was found to be 2.42 (see Figure S3 in Supplementary Materials), which is higher than the theoretical value of 1.5, predicted for the semi-dilute entangled regime, likely indicating concentrated entangled regime [38]. The fact that the exponent of the associative graft copolymer PAA-*g*-PLL is more than two-fold implies that the sharper increase in the viscosity is due to the intermolecular polymer association through ionic complexation. The significant viscosity enhancement of PAA precursor with a concentration in the same concentration regime suggests that besides ionic complexation, chain entanglements also contribute to the elasticity of the formed network.

**Figure 2:**
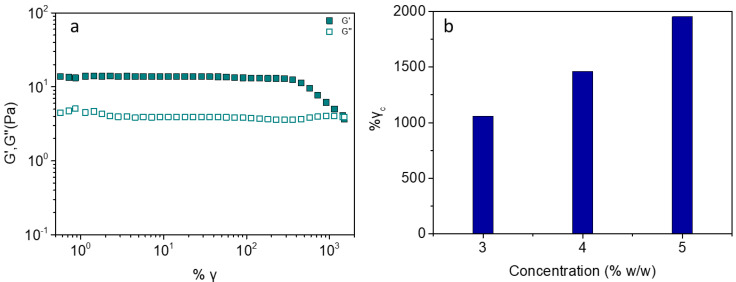
(**a**) Strain sweep (C_p_ = 4 wt%) and (**b**) γ_c_ vs. concentration of PAA-*g*-PLL at 1 Hz, 25 °C and pH = 7.4.

An interesting feature of these hydrogels is the extended linear viscoelastic regime (LVR) they exhibit. Figure 2a demonstrates a characteristic plot of G′, G″ vs. strain, showing that the LVR is extended up to about 300%, implying significant resistance of the structure deformation upon applying shear stress. The critical strain amplitude, γ_c_ at the G′/G″ crossover point occurs at strains above 1000%, beyond which the formulation flows (G″ predominates G′), reflecting substantial structural rearrangements in the system. The critical strain γ_c_ increases linearly with C_p,_ as depicted in Figure 2b. Specifically, γ_c_ increases about two-fold by increasing C_p_ from 3 to 5 wt%. However, the corresponding critical strain amplitude, σ_c_, required to break the structure allowing flow, increases exponentially with C_p_, i.e., from 41 Pa at C_p_ = 3 wt% to 539 Pa at C_p_ = 5 wt%. This reveals strong resistance to flow due to the network structure strengthening upon increasing the elastically active chains, as reflected in the G′ augmentation pointed out in Figure 1c.

**Figure 3:**
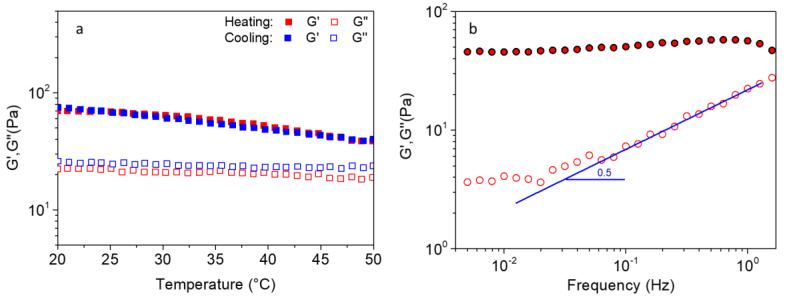
(**a**) Temperature dependance of G′ (closed symbols) and G″ (open symbols) of PAA-*g*-PLL hydrogel sample of C_p_ = 5 wt% at pH = 7.4, f = 1 Hz and γ = 1% with a heating-cooling ramp at 1 °C/min. (**b**) G′ (closed symbols) and G″ (open symbols) vs. frequency of PAA-*g*-PLL hydrogel of C_p_ = 5 wt% (γ = 1%) at pH = 7.4 and 37 °C.

The hydrogel resistance to heat is another important parameter that affects the hydrogel performance. Figure 3 displays G′, G″ as a function of temperature, obtained by a temperature ramp experiment performed on the 5 wt% sample with a heating/cooling rate of 1 °C/min, at a constant frequency of 1 Hz. The moduli decrease smoothly upon heating and recover on cooling practically without hysteresis, revealing good resistance to heat at least up to 37 °C. Figure 3b displays the data from the frequency sweep experiment at the physiological conditions (pH = 7.4, T = 37 °C). As can be observed, the storage modulus predominates to the loss one even in the low-frequency range implying long relaxation times. The same behavior occurs at room temperature and at lower concentrations. Therefore, the dynamics of the ionically associative 3D network, exemplified by the exchange dynamics of the positively charged PLL sequences from the polyelectrolyte complexes, cannot occur in the time scale of the experiment, i.e., at least up to 250 s. This behavior differs remarkably from other hydrogel systems forming 3D networks through the co-assembly of two different copolymers bearing oppositely charged blocks of ABA macromolecular architecture with a non-ionic water-soluble B block. In those cases, and at similar polymer concentrations, the dynamics of the networks are much faster [17,18,39]. A plausible explanation is that in the present system, the L-lysine repeating pendant units encompass a (CH_2_)_4_ hydrophobic spacer between the charged protonated amines and the polypeptide backbone, which might contribute to hydrophobic interactions in the polyelectrolyte crosslinking nanodomains. Another characteristic feature of the viscoelastic response of the hydrogel revealed by the frequency sweep data is that in the high-frequency range, G″ increases again with a power law exponent of 0.5, probably crossing G′ at high frequencies, which is visible at lower frequencies C_P_ < C_gel_. This might be attributed to local movements of the side chains of the graft copolymers. More importantly, gel-like behavior occurs at remarkably higher concentrations in the co-assembling systems [37], which should mainly be attributed to the macromolecular topology of PAA-*g*-PLL and especially to the polyelectrolyte nature of the long PAA chains, as compared to the non-ionic Poly (ethylene oxide) ones.

### 2.2. Effect of pH

**Figure 4:**
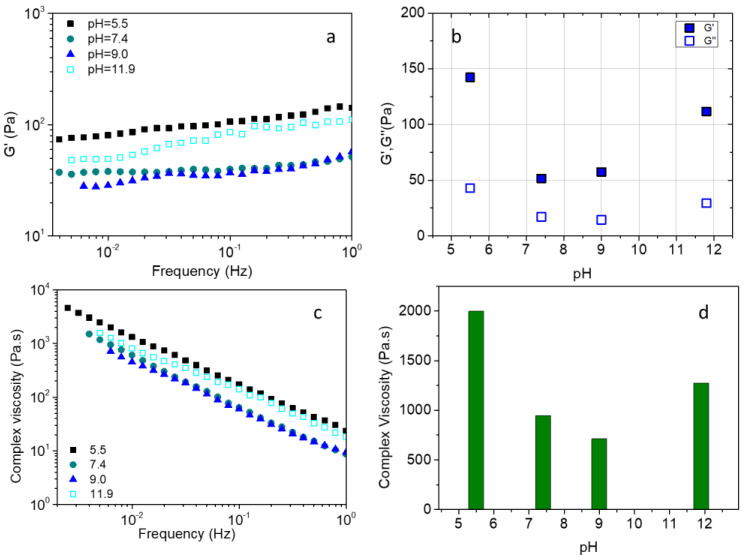
Angular frequency dependance of G′ (**a**) and complex viscosity (**c**) at various pHs of PAA-*g*-PLL hydrogels of Cp = 5 wt%, at fixed strain amplitude 1% (LVR) and 25 °C. pH dependance of G′ (closed symbols), G″ (open symbols) (at 1 Hz) (**b**) and complex viscosity (at 0.006 Hz) (**d**) of samples with Cp = 5 wt% at a fixed strain amplitude 1% (LVR) and 25 °C.

Provided that the gelator is a weak polyampholyte, the environmental pH should affect the network structure and, in turn, the rheological properties of the hydrogel. Hence, salt-free aqueous solutions were prepared at fixed C_P_ = 5 wt% at various pH, and their viscoelastic response was explored by oscillatory shear experiments in the linear regime and at room temperature. Figure 4 depicts the results at four pH values. The frequency sweep data showed a nearly solid-like response in all cases since the storage modulus dominates to loss one in the entire frequency range investigated and varies weakly with frequency (Figure 4a). Similar conclusions arise from the complex viscosity (Figure 4c) vs. frequency plot, showing the absence of any Newtonian behavior at low frequencies but a continuous strong decreasing frequency dependence, revealing shear thinning behavior. In Figure 4b, G′ and G″ at a fixed frequency of 1 Hz have been plotted as a function of pH. As observed, the elastic modulus increases about three-fold by decreasing pH from 7.4 to 5.5 and increases again about two-fold by increasing pH to 11.9. In the intermediate pH 7.4 and 9, similar values were obtained. The data of complex viscosity at low fixed frequencies (Figure 4d) indicate similar behavior.

**Figure 5:**
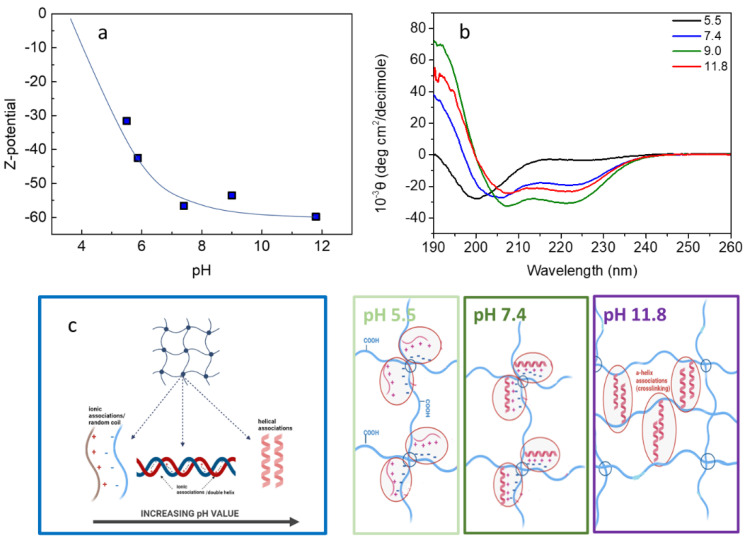
Zeta potential (**a**) and circular dichroism spectra (**b**) of PAA-*g*-PLL aqueous solutions (C_p_ = 0.1 wt%) as a function of pH. (**c**) Schematic representation of the chain conformation of the segments involved in the crosslinking domains and the network structures at various pH.

The variation of pH significantly affects the degree of ionization of the graft copolymer constituents and, in turn, their conformation. Moreover, it influences the strength of ionic interactions between the oppositely charged blocks (PAA-PLL attractive) forming the polyelectrolyte complexes and between similarly charged segments (PAA-PAA repulsive) as well. Specifically, by increasing pH, the PAA backbone ionization degree increases by deprotonation of the carboxylic groups, imposing a conformational transition from random coil to extended rod-like chain conformation [40]. On the contrary, the degree of ionization of the PLL side chains decreases as the amine groups deprotonate, leading to non-ionized chains at very high pH. Therefore, the charge balance exemplified by the [NH_3_^+^]/[COO^−^] molar ratio decreases with increasing pH and tends to zero at pH 12. This is reflected in the variation of the z-potential of the solutions presented in Figure 5a. Provided that the PAA is the major component ([COOH] 92 mol%), the z-potential is negative and decreases with pH as the [NH_3_^+^]/[COO^−^] molar ratio decreases. Thus, in the pH range investigated, the formed network is always of a negatively charged polyelectrolyte nature.

It is well known that the PLL polypeptide is subjected to secondary chain conformational transition from random coil to *α*-helix upon varying pH [41,42]. Therefore, to better understand the influence of pH on the formed structure, circular dichroism (CD) was conducted in dilute solutions at the same pH values. The CD spectra at various pH are gathered in Figure 5b. These spectra give information about the secondary structure of the PLL in the complexes with PAA, which is invisible (no UV adsorption) in this wavelength range.

The CD spectrum at pH 5.5 shows the characteristic pattern of a random coil (single strong negative band at 200 nm) in accordance with the spectrum of pure PLL [43]. At pH 7.4 and 9.0, the two negative bands at 222 and 208 nm (the fingerprint of *α*-helix) reveal a conformation transition from random coil to *α*-helix, contrary to what is observed in pure PLL, exhibiting random coil in this range of pH. However, at pH 7.4, the broadening of the band at 208 nm towards 204 nm should be attributed to the coexistence of some random coil conformations [43]. The random coil to *α*-helix transition occurs at a pH higher than 10.8 (*pKa* of PLL), where the polypeptide becomes uncharged. Indeed, this is valid at pH 11.8, as depicted in Figure 5b. The *α*-helix conformation of the PLL side chains at the intermediate pH should be attributed to the ionic interactions with the negatively charged carboxyl groups of PAA segments that neutralize the positively charged amine groups of PLL stabilizing hence the helical structure. This is in good agreement with the results of Shinoda et al. exploring the complexation between PAA and PLL homopolymers [44]. In the same study, it was indicated that PAA could form a left-handed superhelix bound to the core composed of a right-handed *α*-helix of PLL in the complex, which is reasonable to occur also in our case. Therefore, the main pH effect in the structure of PAA-*g*-PLL networks is the conformational transition of the PLL secondary structure, which affects the association mode of the crosslinks of the network. That is, PAA random coil—PLL random coil (pH 5.5), PAA left-handed superhelix—right-handed a-helix of PLL (pH 7.4, 9.0) and PLL *α*-helix hydrophobic association (pH 11.8) (Figure 5c). These different association modes, together with the repulsive interactions exerted by the non-complexed PAA segments, seem to affect the 3D network structure and especially the number density of the elastically active chains that contribute to the network elasticity as reflected in the pH dependence of the storage modulus G′ (Figure 4c). For instance, by lowering pH to 5.5, the degree of ionization of PAA segments decreases, weakening thus the repulsive interactions between the PAA chains, promoting likely the formation of intermolecular complexes with the pendant PLL charged random coils at the expense of intramolecular ones. This might have also occurred at pH 11.8, where the repulsive interactions should be weakened, due to electrostatic screening from the elevated ionic strength, promoting the intermolecular association of the uncharged PLL *α*-helixes.

**Figure 6:**
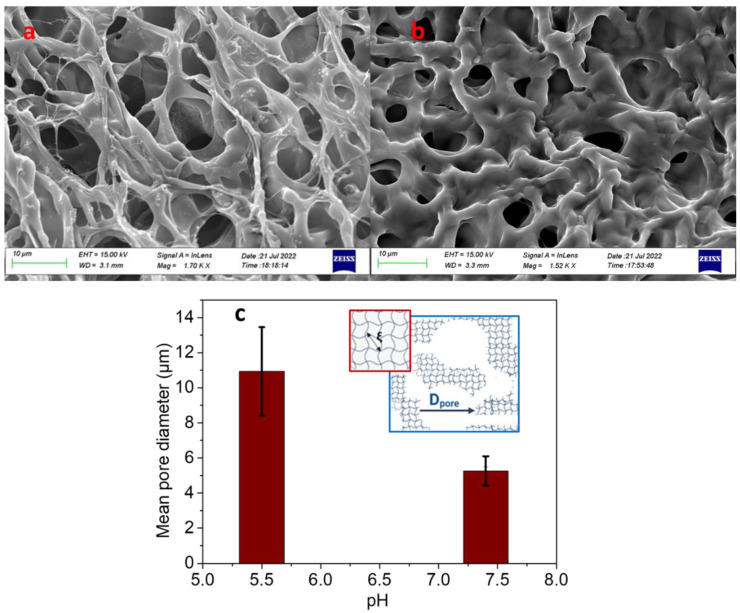
SEM images of the freeze-dried PAA-*g*-PLL hydrogels (Cp = 5 wt%) at pH 5.5 (**a**) and 7.4 (**b**) and their corresponding mean pore size (**c**). The inset demonstrates a schematic network with a typical open pore structure: *D_pore_* is the pore size, and *ξ* is the mesh size of the crosslinked domains.

Scanning electron microscopy (SEM) was performed in two different freeze-dried hydrogels, prepared at pH 7.4 and 5.5 (Cp = 5 wt%), relevant to potential bioapplications. In Figure 6a,b, the micrographs reveal a microporous structure in both cases, differing in the mean pore size (*D_pore_*) (Figure 6c and Figure S4). *D_pore_* increased from pH 7.4 to 5.5, which seems in qualitative agreement with the corresponding increase in the elastic modulus (G′). As it is known, the elastic modulus is inversely proportional to the mesh size *ξ* of the network, G = PKT/ξ (where P is the number of overlapping network strands inside the correlation volume [45], due to the enhancement of physical crosslinks. Therefore, the decrease in *ξ* implies a denser network which seems to lead to a higher *D_pore_* (see schematics in Figure 6c).

### 2.3. Effect of Ionic Strength

**Figure 7:**
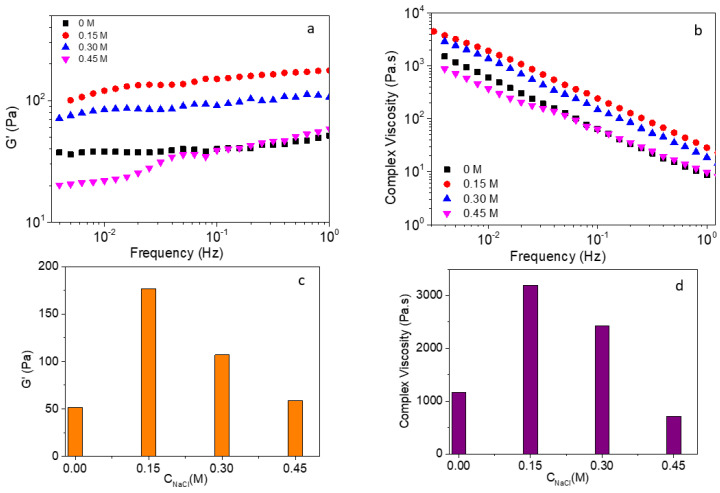
Frequency dependance of (**a**) storage modulus (G′) (**b**) complex viscosity at various salt NaCl concentrations and salt (NaCl) concentration dependance of G′ (at 1 Hz) (**c**) and complex viscosity (at 0.006 Hz) (**d**) of PAA-*g*-PLL hydrogels (C_p_ = 5 wt%, Ph = 7.4, fixed strain amplitude = 1% (LVR) and 25 °C).

Polyelectrolytes and their ionic complexations are sensitive to the ionic strength of the solution. We thus proceeded to explore the influence of the presence of salt on the rheological properties of the hydrogels, prepared at fixed pH 7.4. In all cases, elastic hydrogels were observed as the G′ predominates the G″ in the entire frequency range investigated. In Figure 7, the data (G′, η*) from the oscillatory frequency sweep experiments are demonstrated for various salt concentrations C_NaCl_ up to 0.45 M. The high-frequency G′ increased upon adding 0.15 M NaCl and then decreased steadily upon further increasing C_NaCl_. The initial overshooting of G′ with C_NaCl_ is not rare, as has been observed in other network systems formed by charge-driven assembly [12,34]. At low ionic strength, the repulsive interactions along the PAA chain and among the different copolymers are weakened due to partial electrostatic screening, promoting thus the augmentation of the number density of the intermolecular ionic complexation as reflected in the G′ enhancement. Further increase in C_NaCl_ strengthens the electrostatic screening, increasing hence the chain flexibility of the PAA segments, which seems to impose intermolecular to intramolecular transitions, decreasing G′ eventually.

The same behavior was observed in the complex viscosity, which is affected by the exchange dynamics of PLL from the crosslinks (complexes with PAA) besides the number density of crosslinks. The frequency sweep data do not show the appearance of G′/G″ crossover at low frequencies suggesting that the relaxation of the network is still very slow. In other words, the terminal relaxation time, which is mainly correlated with the lifetime of the crosslinks, is much higher than the time of observation. We speculate that the reduction in the attractive ionic interactions among the oppositely charged segments with increasing ionic strength is compensated by the hydrophobic ones to some extent due to the L-lysine hydrophobic nature, bearing -(CH_2_)_4_ sequences in their pendant groups. This is in qualitative agreement with the results obtained by the co-assembly of PGA-PEG-PGA (PGA is poly(L-glutamic acid) and PLL-PEG-PLL [34].

Another interesting consequence of the effect of increasing ionic strength is the increase in the critical strain γ_c_ above which the formulation flows (G″ > G′). As illustrated in Figure S5, γ_c_ increased remarkably with C_NaCl_. Especially by adding 0.45 M NaCl in the aqueous medium, γ_c_ increased more than two-fold. This might be ascribed to the increase in the chain flexibility of the elastically active PAA segments because of the electrostatic screening of the repulsive interactions along the chain, allowing higher extensibility. Moreover, the weakening of the ionic interactions among the PAA/PLL segments allows easier rearrangements of the crosslinks during the strain evolution.

### 2.4. Shear-Induced Reversibility, Self-Healing and 3D-Printability

**Figure 8:**
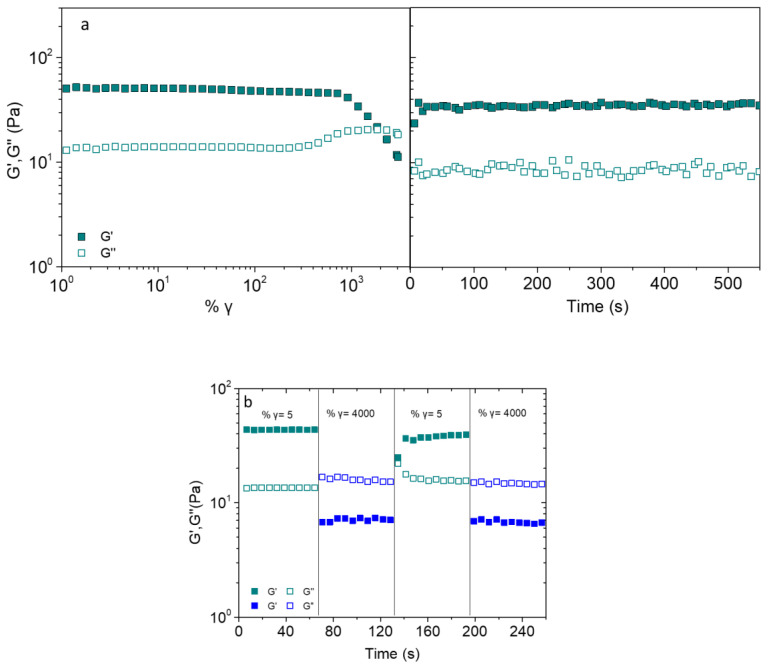
Time sweep of PAA-*g*-PLL hydrogel sample immediately after subjecting to strain sweep up 4000% above γ_c_ (**a**) and consecutive time sweep at fixed strain amplitude: 5% (LVR) and 4000% (beyond LVR) (**b**) of PAA-*g*-PLL hydrogel sample (C_P_ = 5 wt%, pH = 7.4) at 1 Hz and 25 °C.

To explore the self-healing ability of hydrogel, a two-step consecutive experiment was conducted. In the first step, the sample was subjected to a strain sweep beyond the critical strain (at γ = 4000%) to disrupt the 3D structure allowing flow, followed by a second step, setting γ at 5%, within the LVR. As observed in Figure 8a, the 3D structure is recovered instantly as the G′ becomes again higher than G″, exhibiting solid-like behavior. A similar experiment was conducted in shorter time intervals to test the strain responsiveness of the hydrogel. The sample was subjected to a consecutive stepwise time sweep, applying strain amplitudes at 5 and 4000%. As shown in Figure 8b, at γ = 5%, the formulation exhibits gel-like behavior since G′ dominates over G″. Upon a sudden variation of strain at 4000%, the formulation instantly flows as G″ dominates now to G′, implying rupture of the 3D structure as expected (γ > γ_c_). In the third step, by applying low strain again within the LVR, the hydrogel was recovered instantly. A short delay can be observed in the evolution of G′, which is recovered about 90% after 60 s. Finally, in the fourth step, by applying the same strain as the second step, the gel flows again, behaving similarly, which implies very good responsiveness to shear. All the data in Figure 8 clearly indicate that the hydrogel exhibits self-healing with fast recovery after the hydrogel rupture induced at elevated stress. The same behavior was observed in all gelator concentrations (3–5 wt%) and independent of pH or ionic strength.

**Figure 9:**
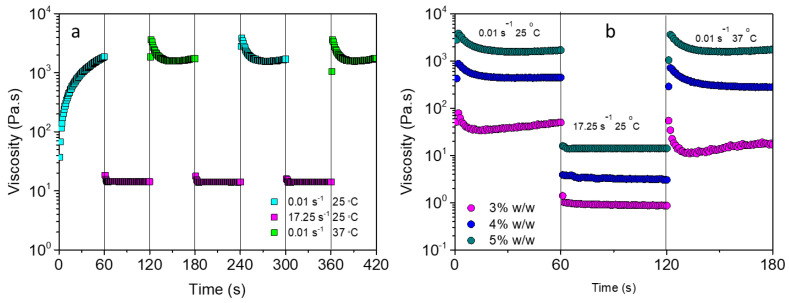
(**a**) Shear viscosity vs. time of 5 wt% PAA-*g*-PLL hydrogel sample subjected to stepwise shear rate and temperature variations: 0.01 s^−1^ and 25 °C (cyan, squares), 17.25 s^−1^ and 25 °C (magenta, squares), 0.01 s^−1^ 37 °C (green, squares). (**b**) Shear viscosity vs. time in three consecutive steps under the same conditions for three polymer concentrations as indicated.

Injectability and 3D printability of hydrogels are important properties relevant to potential bioapplications. To evaluate these properties, steady-state shear flow measurements were conducted. Particularly, in order to estimate the injectability through a syringe needle, consecutive shear viscosity time-sweep stepwise experiments were conducted at room temperature (25 °C) and under shear rates of 0.01 and 17.25 s^−1^ followed by a third step at physiological temperature (37 °C) applying a shear rate of 0.01 s^−1^. The first low-value approach to the zero-shear viscosity, simulates gel at rest, and the second value exemplifies the shear rate applied through a 28-gauge syringe needle [46]. Figure 9a demonstrates time-sweep apparent viscosity data obtained at seven steps under the conditions described previously. As shown, the viscosity drops from 1860 to 14 Pa·s (more than two orders of magnitude) upon applying a sudden variation in shear rate from 0.01 to 17.25 s^−1^ at 25 °C and recovers to the previous values when the shear rate is reverted to 0.01 s^−1^ at 37 °C, simulating conditions after injection at physiological temperature. More importantly, the viscosity changes instantly upon varying the shear rate, implying excellent shear responsiveness of the hydrogel in accordance with the data of the oscillatory measurements (Figure 8). The steps were repeated two times, and the data indicate excellent reproducibility too. The simultaneous sudden change in temperature from 25 to 37 °C with the shear rate changes does not give any effect, showing the appreciable heat resistance of hydrogel again, as we presented previously (Figure 3).

The same behavior was observed for all the formulations of different gelator concentrations, as presented in Figure 9b. Certainly, the viscosity drops with decreasing C_P_ implying that it is a factor that could be used to tune injectability. For instance, at C_P_ = 3 wt%, the viscosity under a shear rate of 17.25 s^−1^ (injection conditions) drops to the order of 1 Pa·s which seems to meet the requirements for injectability. Nevertheless, the shear-induced viscosity decrease demonstrated in Figure 9 suggests that the formulations are at least 3D printable.

**Figure 10:**
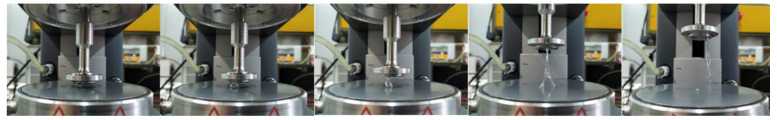
Successive photos showing the adhered formulation during raising the cone of the rheometer.

Another interesting property of the explored PAA-*g*-PLL-based hydrogel is its adhesive capability, which was optically observed on the cone and plate rheometer. As illustrated in the successive images of Figure 10, upon rising the cone, the hydrogel is shown to adhere to the metallic surfaces and resist rupture at high deformations, exhibiting hence appreciable elasticity. The latter seems to be in qualitative agreement with the rheological properties, namely high critical strain γ_c_ (Figure 2), a measure of the resistance of the elasticity to shear deformation.

The PAA-*g*-PLL graft copolymer and its PAA-*g*-P(boc-LL) precursor form hydrogels in aqueous media. These two gelators have the same macromolecular topology, differing only in the nature of the PLL functional repeating units, i.e., NH_2_ (ionogenic) vs. -NH-boc (hydrophobic). Thus, it would be useful to compare their hydrogel properties, provided that they self-assemble in water through different interactions, i.e., ionic vs. hydrophobic. The PAA-*g*-P(boc-LL) hydrogel structure is constituted of densely packed swollen hydrophobically crosslinked finite networks (microgels), while the PAA-*g*-PLL copolymers form a 3D network through ionic complexation. This significant structural difference produces remarkable differences in their rheological properties, namely lower critical gel concentration and critical strain to flow (γ_c_) (about three orders of magnitude) for the PAA-*g*-P(boc-LL), but remarkably higher moduli at comparable polymer concentrations.

## 3. Conclusions

A polyampholyte graft copolymer, constituted of polyacrylic acid (PAA) backbone grafted by PLL, bearing negatively and positively ionogenic moieties, respectively, was designed and evaluated as a gelator in aqueous media. This segmented graft-type polyampholyte self-assembles spontaneously in water mainly through ionic interactions among the oppositely charged segments, forming a 3D network at concentrations above a percolation threshold C_gel_, determined at C_P_ = 2.35 wt%. Dynamic and steady-state shear rheology were used to explore the hydrogel properties. The hydrogels exhibit a nearly elastic response with slow dynamics and remarkable extended critical strain, higher than 1000%, above which the hydrogels flow. Thanks to the weak polyelectrolyte nature of the graft copolymer constituents, the hydrogel exhibits properties sensitive to pH. More importantly, provided that the PLL polypeptide side chains adopt secondary structures affected by the ionic interactions with PAA and/or the medium pH, different network structures were observed. Particularly, upon increasing pH, the association mode of the crosslinks changes from PAA random coil—PLL random coil (pH 5.5) to PAA left-handed superhelix—PLL right-handed a-helix (pH 7.4, 9.0) and finally to PLL *α*-helix association (pH 11.8). In the hydrogel features, we should mention the resistance to heat, the sensitivity to ionic strength and the excellent responsiveness to shear. The latter property provides injectability and 3D printability. Due to physical crosslinks of ionic complexation that can be disrupted under shear deformation and be reformed rapidly at rest, the hydrogel also exhibits self-healing properties. Thanks to the biocompatibility of the copolymer constituents and the biodegradability of PLL, the designed gelator could be considered a good candidate for potential bioapplications.

## 4. Materials and Methods

### 4.1. Materials

PAA-*g*-P(boc-LL) graft copolymer (bearing boc-protected -NH_2_) was synthesized by the grafting onto method using carbodiimide chemistry. Details about this process are reported elsewhere [35]. The graft copolymer molecular characteristics are M_w_ = 575,202 Da and grafting density 8% mol (that is, 11 PLL side chains per backbone). Trifluoroacetic acid (TFA), Hydrochloric acid (HCl, Panreac, Chicago, IL, USA), Sodium Hydroxide (NaOH, Panreac, Chicago, IL, USA), deuterated solvent Deuterium Oxide (D_2_O) (St. Louis, MO, USA) were purchased from Sigma-Aldrich (Athens, Greece) and used without further purification. Ultra-pure three-distilled water (3D-H_2_O) was obtained by means of an ELGA Medica R7/15 device.

### 4.2. Synthesis and Characterization

Simple carbamate hydrolysis in acidic conditions occurred for the removal of boc species from protected amines in the side polypeptide chains. Initially, 0.7518 g (1.30 × 10^−3^ mol) PAA-*g*-P(boc-LL) were weighted and dissolved in 250 mL of 3D-H_2_O and left under stirring for 3 days at 25 °C. A homogenous gel was formed. Afterward, 40 mL of TFA (0.414 mol/in excess) was added and immediately, the gel was transformed into a clear solution. The deprotection reaction was left to proceed for 3 h in a cooling bath due to its exothermic character. The resulting solution was dried by using a Rotary Evaporator to remove the remaining amounts of TFA, and then 200 mL of 3D water was added to dissolve the polymer. To isolate the graft copolymer PAA-*g*-PLL from by-products (Boc moieties and salts), a dialysis procedure in neutral pH against ultra-pure water using a dialysis membrane (MWCO: 12,000 Da) was held for three days. The final PAA-*g*-PLL product was freeze-dried and collected from a vacuum drying oven.

The check of the removal of Boc residues and the characterization of the graft copolymer PAA-*g*-PLL in terms of grafting density was accomplished by Proton Nuclear Magnetic Resonance Spectroscopy (**^1^**H-NMR), using a BRUKER AVANCE III HD PRODIGY ASCEND TM 600 MHz spectrometer (Billerica, MA, USA) at 25 °C.

### 4.3. Preparation of samples for ^1^H-NMR Spectroscopy

An amount of PAA-*g*-PLL graft copolymer was dissolved in deuterated water (D_2_O), and the characteristic ^1^H-NMR spectra are presented in Supplementary Materials (Figure S5).

### 4.4. Hydrogel Sample Preparation

Aqueous solutions of PAA-*g*-PLL graft copolymer were prepared at different concentrations, various pH conditions and salt concentrations, as shown in Table 1. The samples were homogenized using a Vortex mixer machine and a Sigma 2K15 centrifugation machine (2000–5000 rpm and 25 °C) and left to fully dissolve on a lab shaker for one day. The solute for all the samples was phosphate buffer solution (PB) Na_2_HPO_4/_KH_2_PO_4_ (PB) 1 mM and pH = 7.4, and the pH value was controlled by adding HCl or NaOH solution (1M). For examination of the salt effect, the graft copolymer solutions with specific ionic strength were formed by adjusting the NaCl concentration in solute (PB).

### 4.5. Rheology

The rheological studies of the graft copolymer hydrogels and solutions were conducted by using a stress-controlled AR-2000ex (TA Instruments, New Castle, DE, USA) rheometer, with a cone and plate geometry (diameter 20 mm, angle 3°, truncation 111 μm), equipped with a Peltier plate system that allows the precise control of temperature (±0.1 °C) and a solvent trap that prevents changes in concentrations due to water evaporation. The experiments were performed at 25 °C.

### 4.6. Scanning Electron Microscopy

Hydrogel samples (C_p_ = 5% wt) at pH = 5.5 and pH = 7.4 were prepared in the same manner above mentioned in Section 4.4. Then, they were frozen-dried and spattered with gold. SEM micrographs were obtained by a Bruker SEM, LEO-SUPRA VP35 with EDX Microanalysis Unit, at FORTH/ICE-HT in Patras, Greece.

### 4.7. Circular Dichroism

The Circular Dichroism spectra were obtained by using a JASCO J-815 model (Jasco Corporation, Tokyo, Japan). A 0.1 cm cell well was filled with polymer aqueous solutions of different pH (controlled by adding 1 M HCl or NaOH)and at concentrations close to 1.0 × 10^−3^ g mL^−1^.

### 4.8. Zeta Potential

Z-potential measurements of aqueous polymer solutions of different pH were conducted by using a Brookhaven Instruments Nanobrook Omni (Holtsville, NY, USA) system operating at λ = 640 nm and with 40 mW power, with the Smoluchowski method, operating in Electrophoretic Light Scattering (ELS) mode.

## Figures and Tables

**Table 1:** Characteristics of the samples.

Sample	C_P_ (wt%)	pH	Ionic Strength (M)
1	1	7.4	-
2	2	7.4	-
3	3	7.4	-
4	4	7.4	-
5	5	7.4	-
6	5	5.5	-
7	5	9.0	-
8	5	11.9	-
9	5	7.4	0.15
10	5	7.4	0.30
11	5	7.4	0.45

## Data Availability

The data presented in this study are available on request from the corresponding author.

## Supplementary Materials

The following supporting information can be downloaded at: https://www.mdpi.com/article/10.3390/gels9070512/s1, Figure S1. The viscosity of aqueous samples of pure PAA (blue, squares) and graft-copolymer PAA-g-PLL (red, squares) vs. shear rate in the same polymer concentration (5% *w*/*w*) (a) their corresponding strain sweeps at 1 Hz and 25 °C (b) The digital photos (inset of a) indicate the flow of the solution pure PAA and the free-standing gel PAA-g-PLL and punctuate the charge-driven self-assembling network formation; Figure S2. G′ and G″ as a function of the frequency of PAA-g-PLL aqueous solutions (pH 7.4) of different concentrations (as indicated); Figure S3: Zero shear viscosity of aqueous solutions of PAA precursor as a function of concentration at pH= 7.4 and 25 °C; Figure S4: Pore diameter distribution of dried samples PAA-g-PLL (C_p_= 5 wt%) at two different pH with its corresponding Gaussian fitting (a) 5.5, (b) 7.4; Figure S5: γ_c_ (from strain sweep data) vs. NaCl concentration in aqueous solutions of PAA-g-PLL (C_p_ = 5 wt%) at pH = 7.4. Figure S6: 1H NMR spectra of PAA-g-PLL. The mol percentage of the copolymer was calculated from the e (CH_2_/PLL) and f (CH/PAA) bands.
