# Supplementary material for: pH-Sensitive Poly(acrylic acid)-g-poly(L-lysine) Charge-Driven Self-Assembling Hydrogels with 3D-Printability and Self-Healing Properties
Source: Gels. 2023 Jun 25;9(7):512. doi: 10.3390/gels9070512 (PMC10379232; doi:10.3390/gels9070512)
Supplement: Supplementary file 1 [file gels-09-00512-s001.zip › gels-2477671-supplementary.pdf]

gels

MDPI

Article

pH-Sensitive Poly(acrylic acid)-g-poly(L-lysine) Charge-Driven Self-Assembling Hydrogels with 3D-Printability and Self-Healing Properties

Maria-Eleni Kargaki [1], Foteini Arfara [2], Hermis Iatrou [2] and Constantinos Tsitsilianis [1,*]

1 Department of Chemical Engineering, University of Patras, 26500 Patras, Greece; kargakim@upnet.gr
2 Department of Chemistry, University of Athens, Panepistimiopolis, Zografou, 15771 Athens, Greece; a.foteini@gmail.com (F.A.); iatrou@chem.uoa.gr (H.I.)
* Correspondence: ct@chemeng.upatras.gr; Tel.: +30-2610-969531

Supporting Information

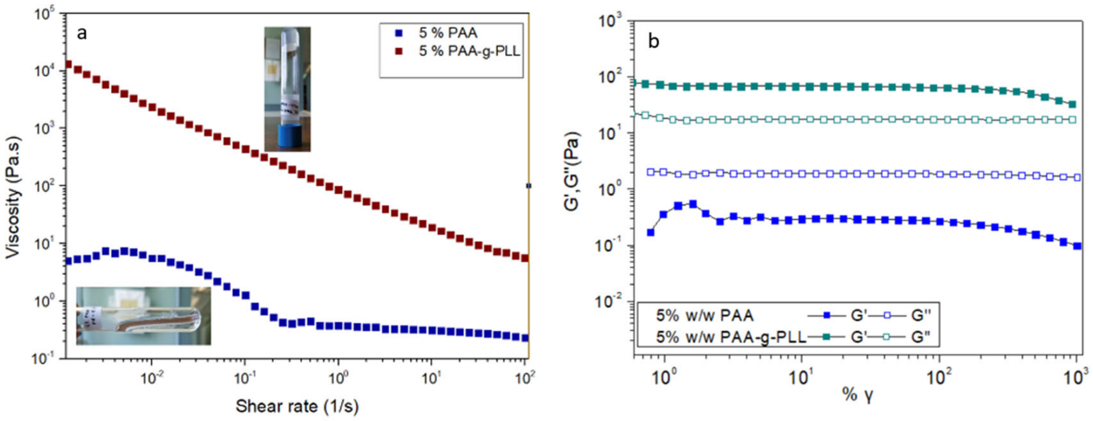

Figure S1. Viscosity of aqueous samples of pure PAA (blue, squares) and graft- copolymer PAA-g-PLL (red, squares) versus shear rate in same polymer concentration (5 % w/w) **(a)** their corresponding strain sweeps at 1 Hz and 25 °C **(b)** The digital photos (inset of a) indicate the flow of the solution pure PAA and the free-standing gel PAA-g-PLL and punctuate the charge-driven self-assembling network formation.

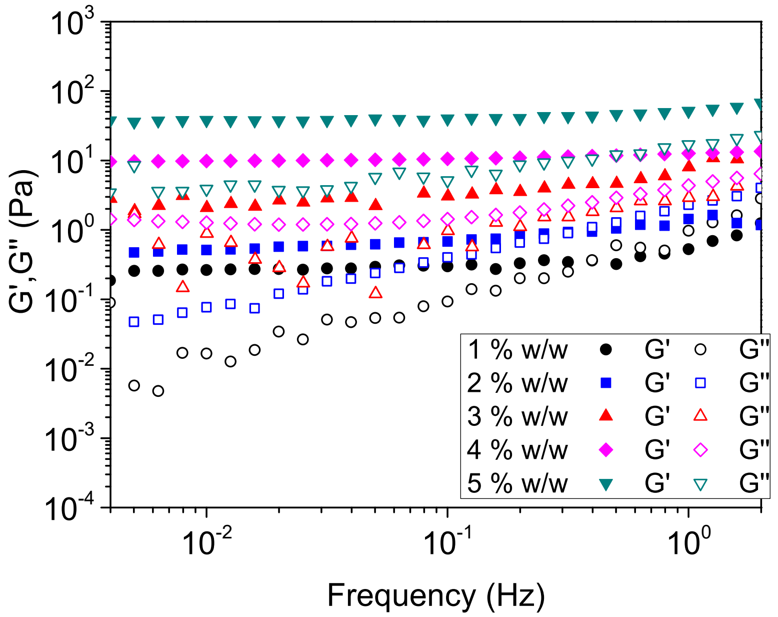

Figure S2. G′ and G″ as a function of frequency of PAA-g-PLL aqueous solutions (pH 7.4) of different concentrations (as indicated).

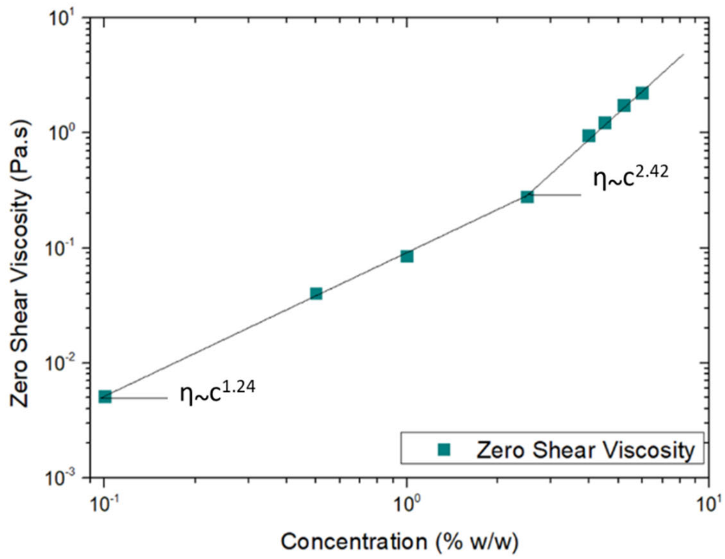

Figure S3. Zero shear viscosity of aqueous solutions of PAA precursor as a function of concentration at pH= 7.4 and 25 ºC.

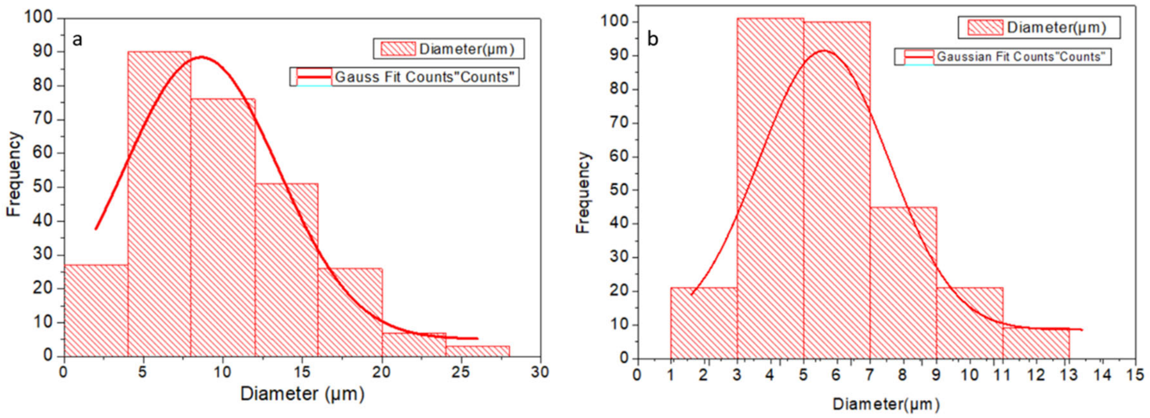

Figure S4. Pore diameter distribution of dried samples PAA-g-PLL (C_P= 5 wt %) at two different pH with its corresponding Gaussian fitting **(a)** 5.5 **(b)** 7.4.

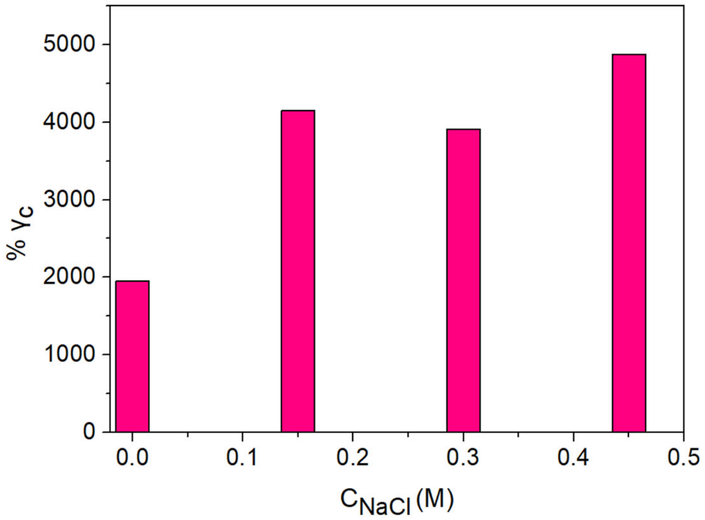

Figure S5. γ_c (from strain sweep data) versus NaCl concentration in aqueous solutions of PAA-g-PLL (Cp = 5 % wt) at pH=7.4.

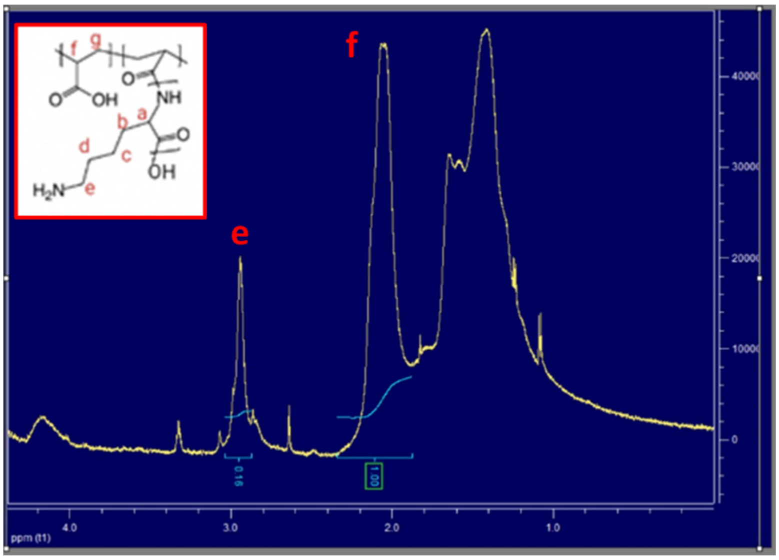

Figure S6. ^{1}H NMR spectra of PAA-g-PLL. The mol percentage of the copolymer was calculated from the e (CH_2/PLL) and f (CH/PAA) bands.
